# Is different better? Models of teaching and their influence on the net financial outcome for general practice teaching posts

**DOI:** 10.1186/1472-6920-11-45

**Published:** 2011-07-12

**Authors:** Caroline O Laurence, Linda E Black, Carolyn Cheah, Jonathan Karnon

**Affiliations:** 1 Adelaide to Outback GP Training Program, 183 Melbourne Street North Adelaide, South Australia, Australia; 2 Discipline of Public Health, University of Adelaide, South Australia, Australia; 3 Discipline of General Practice, University of Adelaide, South Australia, Australia

## Abstract

**Background:**

In Australia, training for general practice (GP) occurs within private practices and their involvement in teaching can have significant financial costs. At the same time there are growing demands for clinical places for all disciplines and for GP there is concern that there are insufficient teaching practices to meet the demand at the medical student, prevocational and vocational training levels. One option to address this may be to change how teaching occurs in the practice. A question that arises in posing such an option is whether different models of teaching change the costs for a teaching practice. The aim of this study is to determine the net financial outcome of teaching models in private GP.

**Methods:**

Modelling the financial implications for a range of teaching options using a costing framework developed from a survey of teaching practices in South Australia. Each option was compared with the traditional model of teaching where one GP supervisor is singularly responsible for one learner. The main outcome measure was net financial outcome per week. Decisions on the model cost parameters were made by the study's Steering Group which comprised of experienced GP supervisors. Four teaching models are presented. Model 1 investigates the gains from teaching multiple same level learners, Models 2 and 3, the benefits of vertically integrated teaching using different permutations, and Model 4 the concept of a GP teacher who undertakes all the teaching.

**Results:**

There was a significant increase in net benefits of Aus$547 per week (95% confidence intervals $459, $668) to the practice when a GP taught two same level learners (Model 1) and when a senior registrar participated in teaching a prevocational doctor (Model 3, Aus$263, 95% confidence intervals $80, $570). For Model 2, a practice could significantly reduce the loss if a registrar was involved in vertically integrated teaching which included the training of a medical student (Aus$551, 95% confidence intervals $419, $718). The GP teacher model resulted in a net remuneration of Aus$207,335 per year, sourced predominantly from the GP teacher activities, with no loss to the practice.

**Conclusions:**

Our study costed teaching options that can maximise the financial outcomes from teaching. The inclusion of GP registrars in the teaching model or the supervisor teaching more than one same level learner results in a greater financial benefit. This gain was achieved through a reduction in supervisor teaching time and the sharing of administrative and teaching activities with GP registrars. We also show that a GP teacher who carries a minimal patient load can be a sustainable option for a practice. Further, the costing framework used for the teaching models presented in this study has the ability to be applied to any number of teaching model permutations.

## Background

In Australia there is an increasing demand for medical clinical placements reflecting the increasing numbers of students being trained [1]. For most medical disciplines the provision of clinical placements is offered through their work within a teaching hospital, but for general practice (GP), this mainly occurs within the community, and in private practice. In addition to the increased volume of trainees requiring placements in GP, there are also changes to the timing and length of placements. For example, several of the new medical schools in Australia require medical students to be placed in general practices from their first year of training, and as they progress through training these student placements will vary in length up to and including 12 months duration depending on curriculum training requirements. Additionally, the introduction of the Prevocational GP Placement Program (PGPPP) now sees an increasing proportion of junior doctors (PGY1- interns, PGY2 and PGY3) undertaking generalist medical rotations in GP as part of their pre-speciality medical training. These placements are in addition to a growing number of registrars, through the Australian General Practice Training Program, who are required to undertake the bulk of their speciality training within GP training environments.

There is growing concern that there are not sufficient practices to accommodate the increasing teaching load at the medical student, prevocational and vocational training levels. One option to address the increased demands for clinical placements in GP is to increase the number of trainees (across the vertical continuum) placed in practices that are already involved in teaching with commensurate support [2]. Another option is to recruit more practices to take up teaching responsibilities. However, Laurence et al [3] has shown that there are significant financial costs associated with teaching in private practice that need to be measured against potential financial benefits when deciding to become a teaching practice. A further option is to change how GP teaching occurs within a private practice. Different models of teaching may increase the teaching capacity of a practice whilst reducing the costs of teaching to a practice at the same time.

The aim of this study is to determine the net financial outcome of various teaching models that currently exist and/or could exist in GP compared with the traditional method of one GP supervisor with singular responsibility for one learner.

### Medical training in General Practice in Australia

Medical students, prevocational doctors (interns and PGY2-3) and vocational level GP registrars in Australia are all trained within general practices, to a varying degree, mostly depending on their regional location. During medical school training, students are placed in general practices at each year of their clinical training with the length varying from one week to 12 months. The Australian Government also funds a number of training posts in GP to conduct prevocational training through the PGPPP [4]. Under this program, Interns (PGY1) and PGY2-3 doctors undertake between a 10 and 12 week or three month rotation respectively in GP. GP vocational training is delivered through 17 Regional Training Providers (RTPs) who each partner with their local network of general practices, all under the auspice of the Australian General Practice Training Program [5]. GP registrars spend a minimum of two out of their three or four year program based in a GP. During this time the registrars will move from junior (GPT1 and GPT2) to senior (GPT3) registrar training levels.

For both PGPPP and GP vocational training there is a minimum amount of scheduled or formal teaching time with the supervisor. The amount varies between the different training levels and are specified in the PGPPP (Practice) Guidelines [6] for junior doctors and the Training Post Standards from the Royal Australian College of General Practitioners [7] for GP registrars. For PGPPP trainees it is a minimum of one hour per week, for GPT1 it is three hours per week and for GPT2 one and half hours per week while there is no minimum required for GPT3. At each of these levels of training some financial subsidies are provided to general practitioners and practices involved in teaching. The amounts vary substantially across the training levels that make up the vertical training continuum. Also distinct stages of training within each level of training, attract differing subsidy amounts.

## Methods

### Teaching models

Three categories of teaching models were developed that reflect current and/or proposed teaching options deemed feasible in GP in Australia. The three categories are described in Table 1.

**Table 1:** Description of the teaching model categories

Category 1	Concurrent teaching of same level-learners: this model facilitates economies of scale from a supervisor integrating their teaching of more than one same level learner
Category 2	Vertically integrated teaching across different levels of learners: these models incorporate vertically integrated teaching where a GP registrar, along with the GP supervisor, participates in teaching

Category 3	A 'GP Teacher' undertaking all required teaching across all levels of learners: this model incorporates all required teaching activity plus a significant percentage of the supervisory responsibility into the 'GP Teacher's' workload with only a minimal independent patient load

From these three categories, four model types have been presented in this paper. The categories and models were developed in consultation with experienced GP supervisors involved in teaching medical students, prevocational doctors and/or vocational registrars within the Adelaide to Outback GP Training Program (AOGP) region [8]. This group of five experts made up the study Steering Group. The role of the Steering Group was to determine the degree of change to each cost parameter if activities were shared or transferred between supervisors and registrars. This was based on their experience with the models within their own practices.

The costing framework reported in a related study [3], undertaken in 2007, was used to prepare the net financial outcome of each teaching model presented in this study. The cost variables included in both analyses were: direct teaching costs, which included preparation time for teaching and additional time teaching added to supervisor's session (incorporating formal teaching and corridor teaching); administrative costs which included staff and GP administration time; and infrastructure costs which included room rental foregone and accommodation rental. In the 2007 study the above variables were used to determine the costs associated with the traditional method of teaching (ie one general practitioner undertaking the singular teaching of one learner) regardless of level, type and number of learners in the practice. The mean times and costs from the previous study formed the basis of the teaching models developed for this study and the results from that study, updated to 2010 prices, are provided in Additional file 1. The net financial outcome of each teaching model in this study has been compared to the net financial outcome of undertaking the same volume of teaching as per each model but using the traditional method of teaching for every learner. The prices for both versions of GP teaching have been updated as of January 2010.

The two key underlying assumptions used in the costing framework for all the teaching models in this study were:

• Teaching time attributed to GP supervisors and GP registrars is converted to income foregone using the agreed consulting level for each doctor level.

• The practice would compensate the GP registrar's loss of income (ie the practice pays the registrar for teaching based on the above agreed income foregone formula).

### Cost parameters

For Model types 1, 2 and 3, the cost parameters were adapted for the GP supervisor in accordance with how much of the teaching responsibilities they varied through economies of scale and/or delegated to the registrar. Specifically for Models 2 and 3, which included registrars in the teaching, the changes to the cost parameters included:

• A transfer of costs to the GP registrar such as teacher up-skilling

• A sharing of costs between the GP supervisor and the GP registrar, such as GP administrative time

• An increase in costs against the GP supervisor for supervisor teaching time with the registrar to accommodate increased support for teaching (that is teach-the-teacher time).

• No change in costs that were not affected by change in teaching profile and personnel, such as staff administrative time or infrastructure costs.

The above adaptations were represented as percentage changes from the model when costed using the traditional method of teaching. These percentage changes were determined by the Steering Group based on their experience with input from AOGP Board members. The details on the cost parameter percentage changes for Model 1-3 are shown in Table 2 and in Table 3 for Model 4.

**Table 2:** Percentage changes in cost parameters from comparison model for each teaching model

Description	Model 1	Model 2			Model 3			
	
	GP supervisor teaching two junior registrars	GP supervisor teaching one junior registrar. Registrar teaching one junior medical student			GP supervisor teaching one senior registrar and one senior medical student. Registrar teaching one intern			
**Cost parameters**	**GP supervisor**	**GP supervisor**		**GP registrar**	**GP supervisor**			**GP registrar**
	**Two junior registrars (GPT1)**	**Junior medical student** **(4^th ^year)**	**Junior registrar (GPT2)**	**Junior medical student** **(4^th ^year)**	**Senior medical student** **(6^th ^year)**	**Intern** **(PGY1)**	**Senior registrar (GPT3)**	**Intern (PGY1)**
	
Preparation time	-25	-75	No change	+100	No change	-75	No change	+100
Additional time to session	-20	-80	+20	+100	No change	-80	+30	+100
Staff Admin time	-30	No change	No change	-	No change	No change	No change	-
GP Admin time	No change	-75	No change	+100	No change	-25	No change	+25
Teacher upskilling time	-50	-100	No change	+100	No change	-100	No change	+100
Infrastructure-room rental foregone and house rental	No change	No change	No change	-	No change	No change	No change	-

GPT1 = the first six month placement for a GP registrar in community general practice and was previously referred to as Basic Term. They are referred to as junior registrars. GPT2 = the second six month placement for a GP registrar in community general practice and was previously referred to as Advanced Term. They are referred to as junior registrars GPT3 = third six month placement for a GP registrar in community general practice and was previously referred to as the first part of a 12 month Subsequent Term. They are referred to as senior registrars. 6th year = a medical student in the last year of medical school at the University of Adelaide and deemed a senior medical student. 4th year = a medical student in their first clinical year at the University of Adelaide and deemed a junior medical student.

**Table 3:** Assumptions, unit costs and benefits and data source for the GP Teacher model

	Assumptions	Unit cost and benefits and data source (Aus$)
**Efficiencies in teaching**	25% efficiencies due to economies of scale by having a GP Teacher	
**GP teacher does all teaching and supervision**	Increased service provision by other general practitioners	
**Sessional work**	GP teacher has small independent patient load-does 3 sessions per week (based on 3.5 hours per session). 80% of billing retained by GP Teacher.	GP hourly rate based on 4 Level B (2 × MBS fee $34.40+ 2 × AMA fee $64) +100% bulk billing item (mean $8.35 of urban and rural location rate). Source: AMA
**Medical students-GP teacher undertakes clinical teaching and patient load**	15 hours medical student teaching per week 7 hours of which involves seeing patients (2 per patients per hour). GP teacher receives 100% of billings	GP hourly rate based on 4 Level B (2 × MBS fee $34.40+ 2 × AMA fee $64) +100% bulk billing item (mean $8.35 of urban and rural location rate): Source: AMA
	Receives all teaching allowance Practice Incentive Payment	$100 per session (3 hours)-max 2 session per day through Medicare Australia
**Interns-GP teacher has teaching and supervisory role**	GP teacher receives 100% of income generated by the interns	Interns-Income per patient $453.38 (based on Item 23 100% fee + bulk billing item) and mean number of patients seen per week from AOGP database
	Receives all teaching allowance from PGPPP	$43,680 per annum (urban) or $38,400 per annum (rural) for PGY1 Source: GPET funding
**GP registrars-GP Teacher has teaching role**	**GP teacher receives teaching allowance and practice component of registrar income**	$32,296 per year for GPT1 Source: GPET funding (GP Registrars)
**Practice**	Practice retains all practice subsidies	Interns-$29,000 per annum GPT1 Registrar-$32,296 per year Source: GPET funding (GP Registrars)

	Practice retains % GP registrar income	45% of income generated by GP registrar GP Registrar income-Income per patient $50.39 (based on Level B (half MBS fee $34.40+ half AMA fee $64 + 100% bulk billing item). Source: AMA

	Practice retains 20% of income generated by the GP teacher from their 3 independent sessions per week	

	Costs of teaching by GP and practice accounted for but with an efficiency saving of teaching time by 25%	

	The practice retaining 30% of income generated by the other general practitioners during the 10 hours they are now no longer involved in teaching (based on only saving 50% of that time)	GP income: hourly rate based on 4 Level B (2 × MBS fee $34.40+ 2 × AMA fee $64) +100% bulk billing item (mean $8.35 of urban and rural location rate). Source: AMA

AMA = Australian Medical Association MBS = Medicare Benefits Schedule GPET = General Practice Education and Training Ltd.

### Statistical analysis

Probabilistic sensitivity analysis (PSA) was undertaken to estimate confidence intervals around the expected costs and benefits for each newly developed model and their comparisons. The PSA involved defining probability distributions to represent the uncertainty around the true value of each input parameter for which confidence intervals were estimated. Given the nature of the parameters-bounded by zero and with a positive skew-log normal distributions were used to represent the uncertainty, with the distribution parameters being informed by the survey responses. A Monte Carlo simulation was then undertaken in which 1,000 iterations of the model were evaluated. Each iteration involved the random sampling of parameter values from the defined probability distributions, which informed a distribution of the financial outcomes for each model of teaching. The 2.5^th ^and 97.5^th ^percentile values from the PSA informed the 95% confidence limits for each training model.

## Results

### Category 1-Model 1-Concurrent teaching of same level learners

Model 1 involves a GP supervisor teaching two junior GP registrars (GPT1) in his/her practice. This model takes into account economies of scale that may occur for the practice and supervisor when integrating the teaching of two registrars at the same level.

In this model the assumption is that some formal teaching would occur jointly with both registrars as well as individual teaching per week. For junior registrars, three hours of teaching per week is required and in this model, joint dedicated teaching would occur 1.5 hours per week and the remaining 1.5 hours would be individual teaching for each registrar. This reduces a supervisor's teaching time from six hours to 4.5 hours per week. Opportunistic teaching would remain the same as if teaching two registrars, although preparation time by the supervisor would decrease by 25%. Staff administration time would reduce by 30% but the GP administrative time would remain the same (for example both registrars would require independent formative assessment). Teacher upskilling would occur only once. There would be no change to infrastructure costs.

The net financial outcome of this concurrent teaching model (Model 1) compared with the traditional method of teaching two independent GP registrars is shown in Table 4. Both versions provide a net financial benefit for the practice for teaching, but Model 1 increases the benefit by Aus$547 per week, which is significant (95% CI $459, $668).

**Table 4:** Net financial outcome per week for various teaching models (Aus$)

	MODEL 1-Concurrent learners			MODEL 2-Vertically integrated teaching			MODEL 3-Vertically integrated teaching		
	
	Model 1 (95% CIs)	Traditional model (95% CIs)	Difference: Model-Traditional (95% CIs)	Model 2 (95% CIs)	Traditional model (95% CIs)	Difference: Model-Traditional (95% CIs)	Model 3 (95% CIs)	Traditional model (95% CIs)	Difference (Model-Traditional) (95% CIs)
**COSTS**									
Direct teaching activities	1781	2243	-462	1315	2659	-1344	2460	3518	-1058
Administrative activities	92	121	-28	73	102	-29	165	160	5
Teacher upskilling	57	113	-57	56	91	-36	99	137	-38
Infrastructure	1493	1493	0	898	898	0	1724	1724	0
**Total costs**	**3424** **(3073, 3903)**	**3971** **(3533, 4569)**	**-547** **(-668, -459)**	**2341** **(2114, 2606)**	**3750** **(3403, 4205)**	**-1409** **(-1732, 1167)**	**4448** **(4129, 4884)**	**5539** **(4924, 6599)**	**-1091** **(-1894, -653)**

**BENEFITS**									
Income to practice	3206	3206	0	658	1516	-858	2140	2968	0
Rental subsidy	80	80	0	40	40	0	148	148	-827
Teacher upskilling payment	92	92	0	46	46	0	62	62	0
Teaching allowance	600	600	0	150	150	0	1739	1739	0
Practice subsidy	448	448	0	112	112	0	577	577	0
**Total benefits**	**4426** **(4161, 4708)**	**4426** **(4161, 4884)**	**0**	**1606** **(1428, 1750)**	**2464** **(2345, 2604)**	**-858**	**4666** **(4120, 4977)**	**5493** **(5317, 5687)**	**-827**

**COST BENEFITS**									
Total benefits	**4426** **(4161, 4708)**	**4426** **(4161, 4884)**	0	**1606** **(1428, 1750)**	**2464** **(2345, 2604)**	**-858** **(-1052, -724)**	**4666** **(4120, 4977)**	**5493** **(5317, 5687)**	**-827** **(-1378, -524)**
Total costs	**3424** **(3073, 3903)**	**3971** **(3533, 4569)**	**-547** **(-668, -459)**	**2341** **(2114, 2606)**	**3750** **(3403, 4205)**	-**1409** **(-1732, 1167)**	**4448** **(4129, 4884)**	**5539** **(4924, 6599)**	**-1091** **(-1894, -653)**
**Net financial outcome**	**1002** **(460**, **1448)**	**456** **(199,976)**	**547(4** **59**, **668)**	**-753(-1102,4** **29**)	**-1286-1763,-917)**	**551(419,** **718**)	**218(-572,736)**	**-45** **(-1102,598)**	**263(80,570)**

Note: totals not exact due to rounding.

### Category 2-Model 2-Vertically integrated teaching across registrar and medical student level

This model is the first of the vertically integrated permutations. Model 2 includes a GP supervisor teaching a junior registrar in their second community term (GPT2) and this registrar teaches a 4th year medical student in the practice.

In this model, the GP supervisor retains some responsibility for the medical student, but at a reduced level. Additionally, the GP supervisor does not attend the teacher upskilling for the medical student, transferring this responsibility to the GP registrar (100%). However, as the GP registrar may be a relatively inexperienced medical teacher, some additional time to the GP registrar teaching sessions may include teacher training. Therefore the GP supervisor's additional time to session for teaching a registrar would increase by 20%. There are no changes to infrastructure costs.

The net financial outcome of this vertically integrated teaching model (Model 2) compared with the traditional method of teaching the same number and type of learners are shown in Table 4. The net financial outcome for both the traditional approach and Model 2 is a net financial loss to the practice from teaching, but Model 2 significantly reduces the loss by Aus$551 per week (95% CI $419, $718). This model reduced the loss to the practice from teaching a junior registrar and a 4th year medical student from -Aus$1286 per week (95% CI -$1763, -$917) to -Aus$753 per week (95% CI -$1102, -$429).

### Category 2-Model 3-Vertically integrated teaching across registrar, intern and medical student level

Model 3 is the second example of a vertically integrated teaching permutation and includes a GP supervisor teaching a senior medical student (6th year) and a senior registrar (GPT3), with the registrar teaching an intern doctor (PGY1).

In this model, the GP supervisor retains full responsibility for teaching the senior medical student and the senior GP registrar. Whilst the supervisor retains a high level of responsibility for the intern, their direct supervisory and teaching responsibilities for the intern are reduced. In this model, the registrar takes on a significant portion of the responsibility for GP administration as well as their in-practice supervision and teaching. On this basis they also attend the teacher upskilling related to intern teaching. The GP supervisor continues to attend teacher upskilling relating to the medical student and registrar, but no longer is required to attend the intern related teaching upskilling. As with the previous model there is a teacher training element for the registrar, adding to a GP supervisor's time with the registrar. There are no changes to infrastructure costs.

The net financial outcome of this vertically integrated teaching model compared with the traditional method involving the same number and type of learners is shown in Table 4. The net financial outcome for Model 3 is a net financial benefit of $218 per week for the practice from teaching while the traditional model is a net financial loss of $45 per week. Model 3 provides an additional net financial benefit of Aus$263 per week for the practice which is significant (95% CI $80, $570).

### Category 3-Model 4 -GP Teacher

Model 4 revolves around the inclusion of a GP Teacher whose main role is to undertake all the teaching and the predominant load of the supervision within a practice, with a minimum independent patient load (see Table 3). This model includes teaching a 4th year medical student, an intern and a junior registrar (GPT1). This model results in the GP Teacher's activities bringing in a net remuneration of Aus$207,335 per year with no loss to the practice (Table 5).

**Table 5:** Per week costs and income/benefits of GP Teacher Model (Aus$)

	GP Teacher	Practice	Total
**TEACHING COSTS**			
**Total costs**	**4240**	**1904**	**6144**
	
**INCOME/BENEFITS**			
Income generated by 'other general practitioners' not required to perform majority of teaching and supervisory roles	**-**	636	
Income generated by GP independent clinical session	1794	448	
Income generated by GP Teacher signing off intern patients	2332	-	
Income generated by GP Teacher teaching medical students with patients	747	-	
Teaching benefits	3354	929	
**Total benefits**	**8228**	**2014**	**10241**
	
**NET INCOME/BENEFIT**	**3987**	**110**	**4097**

Total note exact due to rounding.

## Discussion

This study has compared four innovative models of teaching different level of learners in GP compared to the method of teaching in which each learner receives singular training from one GP supervisor. For Model 1 to 3, there was a significant financial gain to the practice when compared to the traditional method of teaching the same number and type of learners. An increase in the net financial outcome for the practice was found when a GP supervisor taught two same level learners as per Model 1 and when a senior GP registrar participated in teaching a junior doctor as per Model 3. For Model 2, a practice could reduce the loss caused by teaching medical students if they involve the registrar in the student's teaching. Model 4 demonstrated that by utilising current subsidies, identifying teaching efficiencies and maintaining a small independent patient load, a GP teacher could be viable within a teaching practice.

The results of this study builds on work developed by DeWitt [9] who has shown that a practice can reduce the impact of teaching on GP productivity by using a 'wave method' of teaching in a practice. What we have been able to do is progress this thinking by incorporating senior registrars in the teaching 'wave' and further calculate the net financial outcome from such models.

Different models of teaching will suit different settings such as rural community, different practice structures (solo, small or large group practice) as well as different stages of a practice's and practitioner's experience with teaching. Our results provide practices with teaching model options based on a comprehensive costing framework. They have a tool to assist them in their selection of a teaching model that will suit their non-financial needs whilst also maximising their net financial outcome from their involvement with teaching. For example, a supervisor new to teaching may select a simpler model, such as Model 1. As they gain skills in teaching they may then move onto a more complex vertically integrated teaching model such as Model 3 where other learners are teachers.

Using GP registrars in vertically integrated teaching is key to increasing the net financial outcome for a teaching practice. For these models to be successfully implemented, GP registrars will need to be skilled in teaching and be willing to teach. Apart from the recognised 'teach the teacher' element of training that was incorporated into the costing of these models, the RTPs involved in delivering vocational training may also need to consider incorporating a teacher training module into GP training. This recommendation is further supported by the new RACGP Curriculum which formally recognises the importance of teaching skills for registrars [10].

Finally we have demonstrated that the concept of a GP Teacher, who provides all the teaching with a significant proportion of the supervision in a practice, whilst maintaining a small patient load, is both financially sustainable and a realistic option for practices to include in their decision-making with regard to suitable teaching models. This type of model may suit a range of general practitioners including recent graduates who purposefully want a blend of medically related activities in their work through to a GP supervisor who wants to maintain their interest in teaching whilst reducing their clinical patient load.

The costing framework that has been developed and used in this study demonstrates how such a tool can facilitate the cost benefit analysis of different teaching options by identifying the net financial outcomes associated with different permutations within each of the three categories of teaching models (multiple same level-learners; vertically integrated teaching; and 'GP Teacher'). It provides a calculation template that could be adapted to determine the costs and benefits involved in teaching within specific GP environments as well as other specialties and health care providers. Additionally, the development of a teaching cost calculator, based on the costing framework, would allow an individual practice to enter their own data and determine the type of model most suitable for them.

### Limitations

There are limitations with this study. Some of the models developed assume a sufficient space is available in the practice for vertically integrated teaching to occur. This may not be possible for all practices, but where extra room exists, the range of vertically integrated teaching models developed allows a practice to determine the most appropriate model to suit their circumstances.

The assumptions made in developing the models were developed by the study Steering Group consisting of GP Supervisors. While the estimates generally erred on the conservative they may not exactly reflect the circumstances of every teaching practice (eg time spent teaching or on administrative tasks). Also the modelling is based on data obtained from South Australian teaching practices and includes only undergraduate medical student placements. Currently the costing framework used to develop the models is being transferred into a teaching cost calculator. This will allow individual practices to customise the parameters against their preferred model options, which in turn will ensure a more individually relevant net financial outcome. The teaching calculator will also allow for other RTPs and Universities to include their own data and so allow the creation of different permutations based on regionally relevant training levels and stages of training within those levels. Finally, the study focused on the costs and benefits of various teaching models, but did not explore the quality of the teaching provided in each model and if different models provide a different end-product for the trainee. This is an important question to ask in light of the costing analysis and worthy of further investigation.

## Conclusions

This study provides evidence that the current financial incentives to teach within private GP can be further boosted. The models provide teaching options for supervisors and practices that increase the financial benefits of teaching by involving GP registrars in the meeting of teaching responsibilities. and/or by re-ordering the delivery of in-practice teaching and education. This study also provides a basis for informing non-teaching practices who may be considering incorporating teaching into their practice.

## Competing interests

The authors declare that they have no competing interests.

## Authors' contributions

CL participated in the design and coordination of the study, compilation of data, performed most of the analysis and drafted the manuscript. LB participated in the design of the study, contributed to the interpretation of results and critically revised the paper. CC contributed to the interpretation of the results and critically revised the paper. JK performed some analysis, contributed to the interpretation of the results and critically revised the paper. All authors read and approved the final version of the manuscript.

## Pre-publication history

The pre-publication history for this paper can be accessed here:

http://www.biomedcentral.com/1472-6920/11/45/prepub

## Supplementary Material

Additional file 1 **Mean costs and benefits of teaching for 1:1 traditional teaching model which formed the basis of the three teaching models developed, updated to 2010 prices**. Click here for file
