# Supplementary material for: Is different better? Models of teaching and their influence on the net financial outcome for general practice teaching posts
Source: BMC Med Educ. 2011 Jul 12;11:45. doi: 10.1186/1472-6920-11-45 (PMC3146948; doi:10.1186/1472-6920-11-45)
Supplement: Additional file 1 — Mean costs and benefits of teaching for 1:1 traditional teaching model which formed the basis of the three teaching models developed, updated to 2010 prices. [file 1472-6920-11-45-S1.DOCX]

### Additional file 1 – Mean costs and benefits of teaching for 1:1 traditional teaching model which formed the basis of the three teaching models developed, updated to 2010 prices.

|  | **Junior medical student (4th year)** | **Senior medical student (6th year)** | **Intern (PGY1)** | **Junior registrar (GPT1)** | **Junior registrar (GPT2)** | **Senior registrar (GPT3)** |
| --- | --- | --- | --- | --- | --- | --- |
| **COSTS** |  |  |  |  |  |  |
| Direct teaching activities | $1,858 | $1,383 | $1,519 | $1,122 | $801 | $616 |
| Administrative activities | $49 | $42 | $81 | $62 | $54 | $39 |
| Upskilling | $36 | $33 | $38 | $57 | $56 | $66 |
| Other costs | $151 | $203 | $774 | $747 | $747 | $747 |
| **Total costs** | **$2,094** | **$1,661** | **$2,412** | **$1,987** | **$1,658** | **$1,468** |
|  |  |  |  |  |  |  |
| **BENEFITS** |  |  |  |  |  |  |
| Income to practice |  |  | $1,399 | $1,603 | $1,516 | $1,569 |
| Rental subsidy (rural only) |  |  | $108 | $40 | $40 | $40 |
| Upskilling payment |  |  | $15 | $46 | $46 | $46 |
| Teaching allowance | $600 | $950 | $789 | $300 | $150 | $0 |
| Practice subsidy |  |  | $558 | $224 | $112 | $20 |
| **Total benefits** | **$600** | **$950** | **$2,869** | **$2,213** | **$1,864** | **$1,674** |
|  |  |  |  |  |  |  |
| **COST BENEFITS** |  |  |  |  |  |  |
| Total benefits | $600 | $950 | $2,869 | $2,213 | $1,864 | $1,674 |
| Total costs | $2,094 | $1,661 | $2,412 | $1,987 | $1,658 | $1,468 |
| **Net financial outcome** | **-$1,494** | **-$711** | **$457** | **$227** | **$207** | **$206** |
